# Glutamate and its receptors in cancer

**DOI:** 10.1007/s00702-014-1182-6

**Published:** 2014-03-09

**Authors:** Andrzej Stepulak, Radoslaw Rola, Krzysztof Polberg, Chrysanthy Ikonomidou

**Affiliations:** 1 Department of Biochemistry and Molecular Biology, Medical University in Lublin, ul. Chodzki 1, 20-093 Lublin, Poland; 2 Department of Otolaryngology, MSW Hospital, Lublin, Poland; 3 Department of Neurological Surgery, Medical University of Lublin, Lublin, Poland; 4 Department of Physiopathology, Institute of Agricultural Medicine, Lublin, Poland; 5 Section of Child Neurology, Department of Neurology, University of Wisconsin Madison, Madison, USA

**Keywords:** Glutamate receptor, Cancer, Growth factor, Prognosis

## Abstract

Glutamate, a nonessential amino acid, is a major bioenergetic substrate for proliferating normal and neoplastic cells on one hand and an excitatory neurotransmitter that is actively involved in biosynthetic, bioenergetic, metabolic, and oncogenic signaling pathways on the other. It exerts its action through a family of receptors consisting of metabotropic glutamate receptors (mGluRs) and ionotropic glutamate receptors (iGluRs), both of which have been implicated previously in a broad spectrum of acute and chronic neurodegenerative diseases. In this review, we discuss existing data on the role of glutamate as a growth factor for neoplastic cells, the expression of glutamate receptors in various types of benign and malignant neoplasms, and the potential roles that GluRs play in cancer development and progression along with their clinical significance. We conclude that glutamate-related receptors and their signaling pathways may provide novel therapeutic opportunities for a variety of malignant human diseases.

## Introduction

Glutamate (Glu) is one of the key neurotransmitters in the mammalian central nervous system responsible for fast excitatory synaptic signaling in the brain. It affects several physiological processes such us learning, memory or behavior. High levels of Glu resulting in activation of respective receptors lead to neuronal cell death called excitotoxicity that is involved in pathophysiology of various neurodegenerative diseases, epilepsy, hypoxia or stroke (Peng et al. 2011).

To exert its function, glutamate requires apt receptors. Their structure and function were recently reviewed in detail (Lau and Tymianski 2010), thereby we only want to stress that glutamate receptors constitute two main groups: metabotropic glutamate receptors (mGluRs), which belong to the superfamily of G-protein coupled receptors, and ionotropic receptors (iGluRs), which form ion channels. Based on the sequence homology and intracellular signal transduction mechanisms, mGluRs have been classified into three subgroups: mGluR1 and mGluR5, coupled to the phospholipase C, belong to group I; group II consists of mGluR2 and mGluR3, whereas group III contains mGluR4, mGluR6, mGluR7 and mGluR8, which are all negatively coupled to adenylate cyclase. Metabotropic GluRs function as dimers, with two glutamate molecules being required for full receptor activation.

Likewise, the iGluRs are divided into three groups based on structural similarities and named according to the type of synthetic agonist that activates them: *N*-methyl-d-aspartate (NMDA) receptors (NMDARs), amino-3-hydroxy-5-methyl-4-isoxazolepropionate (AMPA) receptors (AMPARs), and 2-carboxy-3-carboxymethyl-4-isopropenylpyrrolidine (kainate, KA) receptors (KARs). NMDARs are assembled from seven subunits: NR1, NR2A/B/C/D, NR3A/B, each one being a product of a separate gene. Alternative splicing results in additional heterogeneity of NMDAR subunits. NMDARs consist of two obligatory NR1 subunits and two of four types of regulatory subunits, NR2A, B, C and D, which assemble as a dimer of dimers. The resulting complex might eventually join with either NR3A or NR3B, which, in such a case, replaces one of the NR2 subunits. Moreover, glycine is a requisite natural agonist for NMDARs (Kew and Kemp 2005; Mayer 2005). For the NMDARs, the NR1 subunit is necessary for calcium conductivity of the channel, while NR2 and NR3 subunits determine electrophysiological and pharmacological properties of the receptor (Traynelis et al. 2010).

AMPA receptors are composed of four subunits: GluR1/2/3/4, that show 70 % of homology while being products of separate genes. Similarly to NMDARs, functional AMPARs are heterotetramers. Within the receptor, the GluR2 subunit plays a critical role in the determination of the Ca^2+^ permeability of the AMPARs. Interestingly, the genomic DNA of the GluR2 subunit contains a code for a glutamine (Q) residue at amino acid 607, but during the process of nuclear RNA-editing glutamine is replaced by arginine (R) in the vast majority of neuronal cells, which results in a very low calcium permeability for receptors containing the GluR2 subunit (Kew and Kemp 2005; Lau and Tymianski 2010; Palmer et al. 2005).

Kainate receptors comprise two types of subunits: GluR5/6/7 and KA1 and KA2, which form homo or heterotetramers. Fully functional homotetrameric receptors are composed of GluR5–7 subunits. KA1/2 homotetramers, even though they combine with the agonist, remain inactive. Functional receptors that consist of KA1/2 subunits have heterotetrameric form in conjunction with GluR5/6/7 subunits. Interestingly, GluR2, GluR5 and GluR6 undergo glutamine/arginine editing (Contractor et al. 2011). Therefore, an opportunity of receptors formation with distinct physicochemical and pharmacological properties exists due to the diversity of ionotropic receptor subunits.

## Glutamate as a growth factor for cancer cells

Glutamate has been proven to regulate proliferation, migration, and survival of neuronal progenitor cells and immature neurons during brain development (Ikonomidou et al. 1999; Komuro and Rakic 1993). The ability of uncontrollable propagation and migration characterizes neoplastic cells as well; therefore, glutamate was suggested as a potential growth factor in tumor development. Support for this hypothesis was provided by studies that showed the generation of neurotoxic quantities of glutamate by glial tumor cells ex vivo (in surgical specimens) (Takano et al. 2001) as well as in vitro in glioma cell lines, where extracellular glutamate concentrations up to 500 μmol/L were demonstrated (Ye and Sontheimer 1999). Follow-up studies confirmed that glutamate stimulates glioma cells proliferation in vivo. Moreover, genetic modifications of C6 rat glioma cells facilitated creation of cell lines that release excessive amounts of Glu along with non-secreting lines. Subsequent xenograft studies proved that experimental tumors created from cells that release high amounts of Glu grow more aggressively than those from non-modified cells (which release moderate amounts of Glu) or from non-secreting cells; this phenomenon was closely related to animals’ overall survival times (Takano et al. 2001). Recent studies showed also that excessive glutamate concentrations (in the range of 100 μM) might be found in the extracellular space at the tumor margin in glioblastoma-bearing patients (de Groot and Sontheimer 2011) resulting in neuronal cell death, which in turn facilitates tumor growth (Rothstein and Brem 2001) and possibly relates to epileptic seizures in glioma patients (Oberndorfer et al. 2002). Interestingly, the latter observation has recently been supported by a study that proved the relationship between increased levels of an excitatory neuropeptide, dynorphin 1–17 that comprises glutamate, and cell death or surgery-related tissue injury in vivo in epilepsy patients (Broderick et al. 2008). Such levels of Glu might arise from glutamate uptake/release systems that are aberrantly expressed and/or activated in glioma cells. It has also been proven that glutamate release in gliomas is, at least in part, mediated by a Na^+^-independent cystine–glutamate exchanger X_C_^−^ system, expressed in glioma cell lines and patient-derived glioma cells (Lyons et al. 2007). Pharmacologic inhibition of this system slows tumor growth and extends survival of tumor-bearing animals (Chung et al. 2005). Importantly, X_C_^−^ system seems to be up-regulated in glioma cells acting in concert with the lack of functional Na^+^-dependent transport systems of the excitatory amino acid transporters (EAATs), which are responsible for glutamate uptake and are amply expressed in nonmalignant glial cells (Lyons et al. 2007; Sontheimer 2008). In glioma cells, EAAT1/2 transporters (especially EAAT2) seem to inversely correlate with the degree of malignancy with almost no expression in GBMs (de Groot and Sontheimer 2011). Accordingly, glioma cells not only release Glu to enhance their highly malignant behavior but also are incapable of its reuptake. More recently, it has also been demonstrated that cells from non-CNS cancers may secrete Glu. MDA-MB-231 (human breast), B16F1 (mouse melanoma) and MATLyLu (rat prostate) cancer cell lines release significant quantities of glutamate into their extracellular environment (Seidlitz et al. 2009). A very recent study reported that prostate cancer-bearing patients have serum glutamate levels directly correlating with Gleason score (≤6 vs. ≥8) and primary prostate cancer aggressiveness (Koochekpour 2013). Glutamate tissue levels in fresh frozen human normal pancreatic tissue, chronic pancreatitis (CP) and pancreatic ductal adenocarcinoma (PDAC) tissues demonstrated a striking increase of glutamate in CP and PDAC samples, suggesting that Glu might serve as a molecular switch that decreases the threshold of k-ras-induced oncogenic signaling and increases the chance of malignant transformation of pancreatic cancer precursor lesions (Herner et al. 2011). Glutamate and synthetic GluR agonists stimulated proliferation of A549 lung cancer cells in vitro (Rzeski et al. 2001; Stepulak et al. 2005, 2007) and invasion of PDAC (Herner et al. 2011). Thereby, released Glu can act as a growth factor and a signal mediator in non-neuronal tumor tissues, in both, autocrine and paracrine fashions.

## Expression of glutamate receptors in cancer cells

A long-standing paradigm suggested that glutamate signaling is limited to the central nervous system. Recently, however this opinion has been changed owing to the studies that proved glutamate receptors’ existence in peripheral organs and neoplastic cells (Hinoi et al. 2004; Kalariti et al. 2005). First studies in the mid-nineties of the last century provided evidence that labeled NMDA agonist ([3H]MK801) couples with CNS tumor cells; moreover, in vitro glutamate application resulted in Ca^2+^ influx into those cells (Ohkuma et al. 1994). Follow-up studies proved the existence of ionotropic GluR1 and GluR4 (Korczak et al. 1995), GluR2/3/4/6/7, KA1 (Yoshioka et al. 1996)], NR1 and NR2C (Casado et al. 1996), or GluR1/2/3/4 in surgical samples of glioblastomas (Ishiuchi et al. 2002) along with various other combinations of subunits in cell lines and CNS tumor samples (Aronica et al. 2001; de Groot et al. 2008). It has been also demonstrated that glutamate receptor subunits are expressed in a variety of cancer cell lines and tumors, originating outside of CNS such as colorectal (Chang et al. 2005; Yoo et al. 2004), pancreatic (Herner et al. 2011), hepatocellular (Li et al. 2012) or gastric cancer (Liu et al. 2007), breast cancer (North et al. 2010b), ovarian cancer (Choi et al. 2012), lung cancers (North et al. 2010a), thyroid cancer (Stepulak et al. 2009), oral squamous cell carcinoma (SCC) (Park et al. 2007), larynx cancer (Stepulak et al. 2011), prostate cancer (Abdul and Hoosein 2005), melanoma (Marin and Chen 2004; Pollock et al. 2003) or osteosarcoma (Kalariti et al. 2004), as well as blood neoplasms such as leukemia and lymphoma (Ganor et al. 2009). Still, the majority of the existing studies describe single receptor or its subunit expression in selected types of cancers while a more detailed analysis of glutamate receptor subunit expression in cancer cell lines or solid tumors remains scarce. Hence, we decided to analyze the presence of iGluRs and mGluRs receptor subunits in several cancer cell lines of neuronal and non-neuronal origin. These studies revealed differential patterns of receptor subunit expression, with some specificity for particular neoplasms (Stepulak et al. 2009, 2011). Interestingly, the NR1 subunit of the NMDA receptor, although present in other cell lines derived from CNS neoplasms, was lacking in glioma cell lines (Stepulak et al. 2009), thereby suggesting that NMDARs are not functional in glioma cells. This observation is supported by previous studies, which showed that four analyzed glioma cell lines and patient-derived gliomas were devoid of NR1 subunit (Lyons et al. 2007) or that its expression was very rare in pediatric GBMs (Brocke et al. 2010).

In contrast to NMDARs, AMPA receptors are abundantly expressed in glioma cells, where they play an important role in glutamate-mediated proliferative signals thus enhancing its malignant phenotype (de Groot and Sontheimer 2011; Sontheimer 2008). As a matter of fact, all AMPA receptor subunits were found in CNS-derived tumors (Aronica et al. 2001; de Groot and Sontheimer 2011; Ishiuchi et al. 2002). Furthermore, the presence or lack of GluR2 subunit seems to be crucial for glioma cells invasion potential. It was observed that the majority of invasive gliomas either lack GluR2 expression (Lyons et al. 2007) or GluR2 is expressed at significantly lower levels, as demonstrated in highly malignant pediatric glioblastomas, ependymomas or medulloblastomas (all WHO III or IV) in contrast to low-grade astrocytomas (WHO I or II) (Brocke et al. 2010). Additionally, RNAi experiments in a low-grade glioma cell line demonstrated that down-regulation of GluR2 expression caused a significant increase of cell proliferation (Beretta et al. 2009). On the other hand, over-expression of edited GluR2 subunit by adenovirus-mediated transfer inhibited migration of glioma cells both in vitro and in vivo (Ishiuchi et al. 2002), proving that Ca^2+^-permeable AMPA receptors are crucial for glioma invasion.

Importantly, the presence of unedited (Q) or edited (R) GluR2 (Q/R site) subunit is critical for calcium permeability of AMPA receptor. Developmentally controlled replacement of the arginine with glutamine at this critical site (Q/R site) during an RNA-editing process renders AMPA receptors permeable (unedited GluR2) or non-permeable to Ca^2+^ (Hollmann and Heinemann 1994; Seeburg 1993). Edited form of GluR2 exists exclusively in the adult brain while unedited forms are present in the fetal brain (Burnashev et al. 1992) and in some cancer cells or tumors (Brocke et al. 2010; Maas et al. 2001; Stepulak et al. 2009; Yoshida et al. 2006). When analyzing GluR2 Q/R-editing status in different cancer cell lines, it has been shown that some of them exclusively express the unedited form of GluR2 (SK-NA-S, neuroblastoma) or both, the edited and the unedited GluR2 forms (MOGGCCM, astrocytoma). Interestingly, unedited GluR2 RNA was found also in SK-LU-1 lung cancer cells (Stepulak et al. 2009), forming highly malignant tumors with a tendency to set distant metastases. This shows that cancer cells could express AMPA receptor forms characteristic for Ca^2+^-permeable fetal cells, which could in turn contribute to cancer cells’ invasion potential, as it has been demonstrated for glioma cells (Ishiuchi et al. 2002). Of particular interest is the fact that glioblastoma-tumor initiating cells express high levels of functional, calcium-permeable AMPA receptors containing GluR1 and GluR4 subunits, when compared with the differentiated tumor cultures consisting of non-stem cells derived from the same tumor tissues (Oh et al. 2012). These finding suggests that functional AMPA receptors can be formed in specific areas of the tumor.

As mentioned above, in contrast to AMPARs, NMDARs expression is reported mainly in peripheral cancers. Moderate to high expression of NR1 subunit of NMDAR has been demonstrated in prostate cancer samples, whereas its expression in normal prostate tissue and benign prostate hyperplasia was very low or absent. Similar expression pattern was found in normal colon or cancer specimens (Abdul and Hoosein 2005). NR1 subunit immunohistochemical reactivity was observed in the majority of small-cell lung (North et al. 2010a) or breast cancer samples, where NR2B subunit was also detected (North et al. 2010b). Different combinations or single subunits of NMDARs were demonstrated in cell lines derived from colon cancer (Stepulak et al. 2009; Yamaguchi et al. 2013), breast cancer (North et al. 2010b; Stepulak et al. 2009), oral cancer (Choi et al. 2004), laryngeal carcinoma (Stepulak et al. 2011), lung cancers (North et al. 2010a; Stepulak et al. 2005, 2007), prostate cancer (Abdul and Hoosein 2005), thyroid cancer (Stepulak et al. 2009) as well as in gastric (Liu et al. 2007; Watanabe et al. 2008), esophageal (Kim et al. 2006), and hepatocellular carcinomas (Yamaguchi et al. 2013).

Interestingly, the expression of kainate receptors in cancer cells has not been extensively studied. The presence of GluR5–7 subunits in human glioneuronal tumors (Aronica et al. 2001), GluR5–7 and KA1 in medulloblastomas, and additionally KA2 subunit in neuroblastoma cell lines (Yoshioka et al. 1996) or retinoblastoma cells (Takeda et al. 2000) has been reported. Genome-wide association studies have recently identified GluR5 expression in hepatocellular carcinoma samples (Li et al. 2012), whereas GluR6 subunits were detected in gastric cancer tissue and gastric cancer cell lines (Wu et al. 2010).

Likewise, our earlier study demonstrated the presence of GluR5 in U343 glioma cells, whereas GluR6, GluR7, KA1 and KA2 subunits were found in all 12 analyzed cancer cell lines, which suggests a role of kainate receptors in metabolism and proliferation of cancer cells (Stepulak et al. 2009). However, when analyzing relative glutamate receptor subunit amounts in cancer cells in comparison to their expression in normal human brain on mRNA level, significant differences were observed. As measured by means of real-time PCR technique, the majority of cancer cell lines expressed either NMDA or AMPA/kainate receptor subunits at much lower levels than the normal human brain (HB). NMDAR NR2B subunit expression level in cancer cell lines was very low compared to HB, with the exception of the human colon adenocarcinoma cell line LS180, which showed an expression level approximating 50 % of the estimated level of NR2B subunit expression in HB. Similarly, a strong expression of GluR4 AMPA receptor subunit was detected in two cell lines: TE671 (rhabdomyosarcoma/medulloblastoma) and RPMI (plasmocytoma), and of GluR6 kainate receptor subunit in SK-NA-S (neuroblastoma) and MOGGCCM (astrocytoma) cell lines at levels comparable to those in the HB. Similar levels of expression of KA2 were found also in human TE671 and the HB (Stepulak et al. 2009).

Glutamate receptor subunits have different expression patterns in pediatric CNS tumors as well. Expression of NR2D, NR3A, KA1, GluR4, mGluR1, mGluR4, mGluR5 and mGluR6 was higher in the high-grade tumors compared to human brain. In low-grade astrocytomas, expression of these glutamate receptor subunits was comparable or lower than in HB (Brocke et al. 2010).

Aforementioned studies provide compelling evidence that glutamate receptors are expressed at higher level in the tumors and neoplastic cell lines of brain origin than in those derived from peripheral cancers. The observation that GluRs subunits, which are poorly represented in the adult brain, are expressed in cancer cells (Stepulak et al. 2009) is of interest as well. On the other hand, it has been shown that the expression of NMDA receptor subunits in the brain varies during development; especially NR2D subunits are present at high levels prenatally in rapidly dividing CNS cells with subsequent decrease postnatally. In adults, NR2D presence is limited to small numbers of cells in selected regions of the brain (Cull-Candy et al. 2001; Waxman and Lynch 2005). Thus, expression of NR2D subunits in all the virtually analyzed cancer cell lines (Stepulak et al. 2009) suggests that the re-expression of NR2D in cancer cells may correlate with their proliferative potential. Interestingly, silencing of NR2D subunit did not influence cancer cells phenotypes. The same was observed for KA2 subunit, also present in all cancer cell lines analyzed (Luksch et al. 2011).

The next important question is whether glutamate receptors are functional in cancer cells. Some observations suggest such a possibility. It has been demonstrated with patch-clamp electrophysiological recordings that glutamate might evoke whole-cell currents in human hypothalamic hamartoma slices immediately after surgical resection (Wu et al. 2005). Similarly, glutamate and NMDARs agonists in the presence of glycine increased membrane-depolarization currents in neuroblastoma cells (North et al. 1997), and glioblastoma, astrocytoma, and oligodendroglioma cells responded to kainate by depolarization of tumor cells in culture or tissue slices (Labrakakis et al. 1998).

It was also demonstrated that iGluRs are active and functional in cancer cells derived from peripheral tumors. An analysis of whole-cell patch-clamp recordings of membrane currents proved that in A549 lung cancer and TE671 (rhabdomyosarcoma/medulloblastoma) cell lines application of glutamate (10 mM) resulted in inward currents that were almost completely blocked by application of NMDA and AMPA receptor antagonists. Interestingly, the evoked currents were small, which is consistent with the low expression of these receptors in examined cancer cells (Stepulak et al. 2009).

Importantly, experimental data have also implicated important role of mGluRs in malignant tumor metabolism and progression. Likewise iGluRs, metabotropic receptors were first detected in tumors of CNS origin such as gliomas (Albasanz et al. 1997; Condorelli et al. 1997), gangliogliomas and dysembryoplastic neuroepithelial tumors (Aronica et al. 2001). Interestingly, mGluR3 receptors were present in almost all of the glioma tumor samples (Nicoletti et al. 2007), including glioma initiating cells (Ciceroni et al. 2008) and glioma cell lines (Nicoletti et al. 2007) with exception of the U343 cell line (Stepulak et al. 2009). In contrast, mGluR1 and mGluR5 were highly represented in the neuronal components of brain tumors (Aronica et al. 2001). In pediatric CNS tumors, the metabotropic glutamate receptor subtypes mGluR1, mGluR2, mGluR4, mGluR5 and mGluR6 were expressed at higher levels in the malignant tumors than in low-grade astrocytomas. Glioblastoma, ependymoma and low-grade astrocytoma all showed low expression levels of mGluR8, whereas expression of mGluR8 was firmly up-regulated in medullo-blastomas (Brocke et al. 2010). Similarly, most of the analyzed medulloblastoma tissue samples and medulloblastoma cell lines displayed the presence of mGluR4 receptors, which inversely correlated with tumor growth (Iacovelli et al. 2006).

Parallel to CNS tumors, mGluRs have been shown to be over-expressed in some types of peripheral cancers and neoplasms. High expression of mGluR1 was reported in primary and metastatic prostate cancers, in contrast to non-cancerous prostate tissues in immunohistochemical analysis (Koochekpour et al. 2012). Moreover, mGluR1 expression displayed a cell type-dependent pattern, being higher in androgen-independent and metastatic cell lines rather than in androgen-sensitive or primary prostate cancer cell lines (Koochekpour et al. 2012). mGluR1/2/3/4/5 expression was demonstrated in both androgen-dependent PC-3 and androgen-independent LNCaP prostate cancer cell lines, whereas mGluR6/8 were present in LNCaP cells only (Pissimissis et al. 2009). mGluR4 was reported to be more specifically expressed in colorectal cancers than in normal tissues (Chang et al. 2005); different mGluR combinations were also demonstrated in colon cancer derived cell lines, including LS180 cell line, where all mGluRs types were detected (Stepulak et al. 2009). Given the fact that mGluR4 mediates 5-fluorouracil resistance in human colon cancer cells, which is a major obstacle in chemotherapy of this cancer type (Yoo et al. 2004), it seems that the presence of mGluR4 in some cancers could have functional significance. mGluR4 was also present in the 50 % of immunohistologically analyzed laryngeal carcinomas, with lower expression in stomach, gall bladder and pancreas adenocarcinomas (17–33 %). Very low presence of mGluR4 was demonstrated in thyroid, adrenal glands, and kidney cancers (8–13 %), whereas it was not detected in esophageal, endometrial and prostate cancers, as well as in neoplasms derived from salivary glands and testis (Chang et al. 2005). Single studies presented an expression of different mGluRs in several cancer cell lines, including those originating from thyroid and breast cancers and blood malignancies (Stepulak et al. 2009), laryngeal carcinomas (Stepulak et al. 2011), and osteosarcomas (Kalariti et al. 2004).

Recent studies proved that mGluR1 are responsible for cell growth regulation in breast (Speyer et al. 2012) and renal cancer cells (Martino et al. 2013), both in vitro and in vivo; simultaneously their presence was not found in oral cancer tissues and cell lines, in contrast to mGluR5, which was present in the majority of oral cancer specimens and weakly in adjacent dysplastic oral mucosa (Park et al. 2007). Moreover, melanoma development was connected with the presence of mGluR1 receptors (Ohtani et al. 2008), a finding that was supported by observations showing its expression in melanoma cell lines and melanoma samples, but not in normal melanocytes and benign nevi (Pollock et al. 2003).

Taken all together, considerable evidence exists for glutamate receptors expression in a variety of tumors and cancer cell lines along with proofs that they are functional, thereby might play an important role in neoplastic transformation and cancer progression.

## The role of GluRs in cancer

To substantiate the hypothesis that glutamate receptors are functionally important for tumor growth, several studies evaluated their involvement in tumorigenesis and subsequently proved that at least some of the GluRs might have oncogenic properties. The impact of metabotropic glutamate receptors on tumor growth was highlighted by a series of experiments which showed causal relationship between mGluR1 expression and melanoma development. Chen’s group was the first to demonstrate that ectopic expression of mGluR1 in melanocytes, which normally lack this receptor, was sufficient to induce transformation to malignant melanoma in vivo (Pollock et al. 2003). In subsequent studies, which implemented an inducible siRNA system, they were able to show that mGluR1 was indispensable for the maintenance of transformation of immortalized melanocytes into tumors with short 3–5 days latency. Their tumorigenic potential in both immunodeficient nude and syngenic mice suggested that the immune system does not influence either tumors formation or distant intestine and muscle metastases formation (Shin et al. 2008). Direct evidences for mGluR1-driven melanoma formation and progression were provided in the same year by Ohtani and coworkers who showed that mGluR1 conditionally expressed in melanocyte-induced pigmented lesions at the first stage, followed by appearance of melanoma tumors 52 weeks after transgene activation. When the transgene was inactivated, melanoma growth was inhibited as compared to animals bearing tumors with persistent mGluR1 expression (Ohtani et al. 2008). Similar findings were reported for mGluR5 transgene activation in mice, resulting in skin hyperpigmentation, seconded by melanoma tumor formation with metastases or primary melanoma lesions detected in lymph nodes, lungs, spleen, liver, uvea and meninges that eventually penetrated into the skull bones (Choi et al. 2011). Very recently, oncogenic mGluR1 properties were also attributed to epithelial cells. Mouse kidney epithelial cells displayed in vivo tumorigenicity, when transfected to ectopically express functional mGluR1, resulting in tumor formation in nude mice. Parallel, siRNA-mediated inhibition of mGluR1 expression in renal cancer cells impaired tumor growth in vivo, thus suggesting that sustained expression of mGluR1 is necessary for neoplastic transformation and tumor progression (Martino et al. 2013), whereas targeting mGluR1 gene using shRNA-expressing lentiviral construct reduced growth of breast cancer cells both in vitro and in vivo (Speyer et al. 2012).

Changes in the levels of expression of ionotropic glutamate receptors or their single subunits in experimental conditions were also demonstrated to be important for cancer cells proliferation and invasion, which suggests the involvement of GluRs in cancer progression. As demonstrated in knockout experiments, diminished expression of GluR1 (AMPAR) subunit at mRNA and protein levels inhibited proliferation of glioma cells in vitro and in vivo (de Groot et al. 2008). Another study presented that RNAi-mediated suppression of GluR1 or GluR2 did not affect pancreatic cancer cell growth, however significantly decreased invasion in vitro, and inhibited tumor cell settling in a mouse model in vivo (Herner et al. 2011). Similar, knock-down of the GluR3 gene reduced proliferation and migration, as well as enhanced apoptosis of pancreatic cancer cells, while over-expression of this gene was reported to have opposite effect in vitro and in a subcutaneous xenograft model (Ripka et al. 2010). In contrast, the silencing of GluR2 by siRNA transfection increased glioma cell proliferation (Beretta et al. 2009), whereas gene silencing of GluR4 modulated the mRNA expression of various tumor-suppressor genes, oncogenes and other genes involved in invasion, adhesion and metastatic capabilities, which resulted in significant increase of cell viability of human rhabdomyosarcoma/medulloblastoma (TE671) and human multiple myeloma RPMI8226 cells. Additionally, silencing of GluR4 stimulated migration of TE671 cells (Luksch et al. 2011). Similarly to AMPAR subunits, modulation of expression of genes for NMDAR subunits influenced behavior of cancer cells. Silencing the NR2A subunit-targeted gene inhibited gastric cancer cells proliferation and cell cycle progression resulting in increased proportion of cells in G1 phase (Watanabe et al. 2008). TE671 and A549 lung cancer cells demonstrated reduced cell viability after transfection and specific knockdown of NR1 gene (Luksch et al. 2011).

Importantly, the expression of GluRs as well as their function in cancer development and progression is influenced by genomic and epigenetic modifications resulting in aberrant posttranscriptional processing. Causative for the aberrant cellular function of glutamate receptors in cancer are changes in genomic sequences for mGluRs and selected subunits of iGluRs. Of particular importance is the notion that the presence of rearranged or mutated forms of glutamate receptor subunits might activate cancer cell growth. As mentioned above, insertional mutagenesis of an ectopically expressed mGluR1 in mouse results in melanoma development (Pollock et al. 2003). Likewise, somatic mutations within mGluR3 gene result in an activation of GPCR-mediated mitogen-activated protein kinase 1/2 signaling that results in a transformed cells’ phenotype, which renders an increased migration of melanoma cells along with a loss of anchorage dependency in growth regulation (Prickett et al. 2011). Moreover, a very recent study highlighted the importance of naturally occurring *GRM1* somatic mutations for mGluR1 surface expression, altered basal and agonist-dependent activity, and disruption of intracellular signaling pathways downstream of the receptor, including inositol phosphate (IP) formation, and altered ERK1/2 kinases activity (Esseltine et al. 2013). Since these mutations were identified in different types of neoplasms including lung adeno- and SCC (Kan et al. 2010), colorectal cancers (Sjoblom et al. 2006), and glioblastoma (Parsons et al. 2008), it has been hypothesized that they are relevant and contribute to a cancer phenotype (Esseltine et al. 2013). Clinical genetic analysis of *GRM1* showed that single nucleotide polymorphism of the C allele of rs362962 (coding mGluR1) contributes to human melanoma susceptibility, especially in a subgroup of patients with a low level of sun exposure and tumors located on the trunk and extremities (Ortiz et al. 2007). A similar study performed in women carrying breast cancer revealed a significant correlation between the *GRM1* CC genotype of rs362962 and the development of hormone receptor-negative breast cancer and association of rs6923492 and rs362962 genotypes with age at diagnosis (Mehta et al. 2013).

In contrast to metabotropic receptors, somatic mutations of iGluR subunits in cancers were scarcely investigated. Whole-exome sequencing analysis revealed moderate to high prevalence of somatic mutations in genes coding NR2A, and NR1 subunits of NMDA receptors in melanoma (Wei et al. 2011); however, their possible consequences are not known (Prickett and Samuels 2012).

Nonetheless, in addition to genetic rearrangements, epigenetic alterations seem to play an important role in cancer development and progression. Human cancers are characterized by a global impairment of DNA methylation. Still, hypermethylation of some DNA regions, especially at the promoter CpG islands of tumor-suppressor genes, is observed (Virani et al. 2012). In this context, considerable interests were demonstrated regarding methylation status of NMDAR subunits: NR2A and NR2B promoters. Aberrant promoter CpG islands hypermethylation of *GRIN2B* (NR2B coding gene) during breast cancer progression was reported, showing higher methylation levels and frequencies in ductal carcinoma in situ when compared with preinvasive lesions such as flat epithelial atypia or atypical ductal hyperplasia; significantly higher methylation frequencies in grade III than in grade I of invasive ductal carcinoma have also been shown which suggests that CpG island methylation of *GRIN2B* might be an early event in breast cancer progression (Park et al. 2011). Other groups, on the other hand, found that NR2B promoter methylation exhibits tumor-suppressive activity in human esophageal (Kim et al. 2006) and gastric cancers (Liu et al. 2007), as well as in non-small cell lung carcinoma (Tamura et al. 2011). Aberrant methylation status of NR2B promoter was present in more than 60 % of human gastric and non-small cell lung carcinoma samples, whereas the GRIN2B methylation status alterations were found in no more than 5 % of corresponding normal tissues (Liu et al. 2007; Tamura et al. 2011). Interestingly, gene methylation of NR2B displayed an inverse correlation with gene (Kim et al. 2006) or protein (Tamura et al. 2011) expression, suggesting that NR2B inactivation occurs mainly through epigenetic events (Kim et al. 2006). Moreover, reintroduction of this gene in esophageal cancer or forced expression in gastric cancer cell lines was accompanied by apoptosis or inhibited cell colony formation, respectively, suggesting tumor-suppressor activity for NR2B (Kim et al. 2006; Liu et al. 2007). Recently, the same research group demonstrated similar results for NR2A subunit in colorectal cancers (Kim et al. 2008). In a more clinically oriented analysis, NR2B methylation was significantly associated with a better prognosis regarding survival of patients with SCC rather than those with adenocarcinoma (Tamura et al. 2011). Therefore, rearrangements of glutamate receptors at different genetic and epigenetic levels seem to play a distinct role in their expression and function. Despite the fact that the issue at hand requires more extensive studies, one may already hypothesize that different modifications of GluRs and their respective genes exist in cancer cells, as demonstrated recently by the discovery of new spliced variants of human GRM1 gene in melanoma cells (DiRaddo et al. 2013).

## Potential clinical significance of GluRs in cancer

The unequivocally proven role of GluRs in oncogenesis turned attention towards their potential clinical significance in different types of tumors. Thus, in the clinical settings the expression of glutamate receptors might influence histological differentiation, clinical tumor staging, the presence of metastases and/or overall patient survival rate. One of the key features that distinguish various tumors relates to their histological differentiation and histological signs of malignancy, classified as tumor grading that influences tumor’s malignancy potential and its clinical course. It has been reported that glutamate receptors’ expression is associated with differentiation status in a variety of tumor subtypes. In pancreatic cancer, precursor lesions as well as pancreatic intraepithelial neoplasia AMPAR GluR1 subunit levels were increased in a step-wise manner, suggesting glutamate involvement in a malignant transformation. On the other hand, however, the expression of GluR1, GluR2 and GluR4 subunits was down-regulated in PDAC (Herner et al. 2011). Moreover, in other tumor types a direct relationship between the degree of malignancy and GluRs expression was found. In brain tumors, GluR1 subunit was differentially expressed according to the tumor grading, being elevated in glioblastomas when compared with anaplastic astrocytomas and low-grade astrocytomas, hence correlating with tumor aggressiveness (de Groot et al. 2008). Opposite association was observed when expression of GluR2 was analyzed, which proved to be present in slow-growing GBM-derived tumor stem cells (GBM TSCs) and low-grade tumor samples but not in fast-growing gliomas or high-grade tumor specimens, indicating that GluR2 expression is associated with a low degree of malignancy (Beretta et al. 2009).

Likewise, immunohistochemistry with an anti-GluR2 antibody showed a significant difference in the positivity of staining that was uniformly present in virtually 100 % of benign secretory prostatic epithelium when compared with a high-grade prostatic intraepithelial neoplasia and low Gleason-patterned carcinomas where scarcely any or very low immunoreactivity of GluR2 was observed. This suggests that the presence of GluR2 in benign glands, including post-atrophic and adenosis-type ones, readily distinguishes them from prostate cancer (Hechtman et al. 2012). An opposite pattern was observed in normal oral mucosa showing very weak expression of NR1 subunit of NMDAR, whereas majority of analyzed oral SCC specimens expressed this subunit; albeit the presence of NR1 did not correlate with histological grading of this cancer type (Choi et al. 2004). Another study that implemented human tissue microarrays revealed that NMDAR subunit NR2B expression was associated with the HER2-positive breast cancer subtype, in contrast to the luminal subtype, where NR2B expression was not observed (Li and Hanahan 2013). In medulloblastoma, mGlu4 receptor immunoreactivity highly correlated with the histological features showing decreasing expression levels of this receptor in the following rank order: nodular desmoplastic > classic ≫ large-cell anaplastic tumors (Iacovelli et al. 2006).

Notably, cancer progression results in tumor growth and local or distant metastases development. The relationship between GluRs expression and aforementioned clinical features of cancers has been scarcely investigated though. Only a few reports demonstrated that the tumor size, presence of lymph node metastases and cancer stage were significantly related to high NR1 (Choi et al. 2004) and mGluR5 (Park et al. 2007) expression in oral SCC, whereas significantly lower expression levels of mGlu4 receptors were correlated with spinal cord metastases, CSF spreading, and recurrence of medulloblastoma (Iacovelli et al. 2006).

Tumor progression along with its dissemination and recurrence are closely related to patients’ prognosis and overall survival. It has been demonstrated that NR1 subunit expression was associated with unfavorable outcome in patients with oral SCC (Choi et al. 2004), whereas mGluR5 expression showed positive correlation with an overall survival of patients with the same malignancy (Park et al. 2007). Similarly, in medulloblastoma the expression of mGluR4 was higher in patients, who survived 5-year after surgery (Iacovelli et al. 2006). On the contrary, overexpression of mGluR4 is associated with a poor prognosis in colorectal carcinoma (Chang et al. 2005). Interestingly, GluR2 expression showed a significant correlation with longer progression-free and overall survivals and was down-regulated in chemoresistant tumors, proving to be a positive prognostic factor for patients with advanced serous papillary ovarian adenocarcinoma (Choi et al. 2012). Gene expression analysis of several hundred glioblastoma samples revealed that a loss of GRIA2 (gene for GluR2) expression was 1 of the 38 gene changes that predict a poor prognosis in glioblastoma (Colman et al. 2010).

Whenever high NR2B expression levels correlated with high vGluT2 vesicular glutamate transporter expression, the survival of patients bearing glioblastoma was significantly shorter, when compared with the patients groups that expressed low levels of NR2B/vGLUT2 (Li and Hanahan 2013).

## Concluding remarks

Research into the role of Glu signaling in cancer development and progression is still in its infancy; however, important progress has been made in recent years. Considerable evidence exists and indicates that glutamate plays an important role in tumor development, acting as a growth factor and a signal mediator in neural as well as non-neuronal tumors tissues, in both autocrine and paracrine fashions. It has been proven that its actions involve mainly a family of receptors consisting of metabotropic glutamate receptors and ionotropic glutamate receptors whose presence has been proven in a variety of benign and malignant lesions throughout the body. Their actions, however, might differ significantly due to the fact that GluRs are combined from various subunits, which result in a large diversity of intracellular signaling and distinct pharmacological properties. Moreover, receptor subunits such as the *NR1* subunit of the NMDA receptor are prone to post-transcriptional events (RNA editing and alternative splicing of RNA), which may result in distinct isoforms (for example eight in case of NR1 subunit) due to the presence of independent sites of alternative splicing. On top of that receptor subunits might undergo further posttranslational and epigenetic modifications resulting in even more complex glutamate signal transduction. Thus, the clinical significance of glutamate receptor expression may differ among tumor entities and it is difficult to predict how the expression of a particular subunit will influence cancer behavior. Still, it is tempting to speculate that GluRs and their signaling pathways render promising targets for therapeutic interventions. As a matter of fact, in recent years, there have been multiple attempts to implement iGluR and mGluR antagonists, in particular in malignant glioma treatment. Drugs such as AMPAR/KR inhibitors, ZK 200775 and GYKI 52466, despite having little effect on glioma growth in vitro, have been shown to exert anti-proliferative and anti-excitotoxic effects in rat hippocampal glioma models. Similar results were obtained for the NMDAR antagonists, norketamine 72 and MK801 (memantine). In fact, memantine has even been employed in a Phase II clinical trial to determine its safety/efficacy in glioma patients; however, results are not yet available (http://Clinicaltrials.gov #NCT01260467). Similarly, the effectiveness of talampanel (an AMPAR antagonist) against glioma has been explored in the clinic. This study was completed with results that were not encouraging though (de Groot and Sontheimer 2011). Interestingly, despite all of the preclinical work and clinical trials (in progress or completed) involving iGluR antagonists, studies targeting mGluR in glioma treatment are lacking. mGluRs, being able to form functional homodimers as typical GPCRs, are considered more susceptible to anti-cancer drug design (Willard and Koochekpour 2013) and may constitute better drug targets than iGluRs (Teh and Chen 2012). Yet, no clinical trials are currently ongoing. Future studies in this field are clearly needed to determine the efficacy of mGluR antagonists and Glu release inhibitors such as riluzole against tumors.

It is clear that more research is needed to define the clinical significance of Glu–GluR expression and signaling in various cancers. Given existing preliminary studies, it will be extremely interesting to follow the field of glutamatergic signaling in cancer in future years.
